# Contrast-enhanced ultrasound in detecting wall invasion and differentiating bland from tumor thrombus during robot-assisted inferior vena cava thrombectomy for renal cell carcinoma

**DOI:** 10.1186/s40644-019-0265-x

**Published:** 2019-12-02

**Authors:** Qiu-Yang Li, Nan Li, Qing-Bo Huang, Yu-Kun Luo, Bao-Jun Wang, Ai-Tao Guo, Xin Ma, Xu Zhang, Jie Tang

**Affiliations:** 1 Department of Ultrasound, Chinese PLA General Hospital, Beijing, 100853 China; 2 Department of Urology, Chinese PLA General Hospital, Beijing, 100853 China; 3 Department of Pathology, Chinese PLA General Hospital, Beijing, 100853 China

**Keywords:** Intraoperative contrast-enhanced ultrasound, Robotics, Thrombectomy, Renal cell carcinoma, Inferior vena cava

## Abstract

**Background:**

Vena cava thrombus is one of the main clinical manifestations of locally aggressive renal cell carcinoma (RCC). Inferior vena cava (IVC) wall invasion and presence of bland thrombus could affect the surgical outcome. This study aims to assess the value of contrast-enhanced ultrasound (CEUS) in detecting wall invasion and differentiating bland thrombus from tumor thrombus during robot-assisted IVC thrombectomy for RCC.

**Methods:**

The intraoperative CEUS findings of 60 patients with RCC accompanied by IVC tumor thrombus were retrospectively analyzed. The CEUS features were compared with the intra- and post-operative pathological findings. CEUS in patients with wall invasion showed that the tumor thrombus was enhanced synchronously with the IVC wall, and the continuity of the IVC wall was lost. In contrast, in patients without wall invasion, CEUS showed that the contrast agent could pass between the tumor thrombus and the IVC wall, and the continuity of IVC wall was good. Typically, contrast-enhanced perfusion was seen in tumor thrombus but not in bland thrombus. The sensitivity, specificity, positive predictive value, negative predictive value, and accuracy of CEUS were statistically analyzed.

**Results:**

The sensitivity, specificity, accuracy, positive predictive value, and negative predictive value of the typical enhancement mode of CEUS were 93.1, 93.5, 93.3, 93.1, and 93.5% in identifying wall invasion and 100, 96, 96.7, 83.3, and 100% in differentiating bland thrombus from tumor thrombus, respectively. There were excellent inter-observer agreements for identifying IVC wall invasion and differentiating bland thrombus from tumor thrombus with kappa coefficients of 0.90 and 0.97.

**Conclusions:**

The present study indicates that intraoperative CEUS plays an important role in robot-assisted IVC thrombectomy for RCC. It can detect wall invasion and differentiate bland thrombus from tumor thrombus, thus offering real-time information to the operator during surgery.

## Background

Vena cava thrombus is one of the main clinical manifestations of locally aggressive renal cell carcinoma (RCC). Approximately 4–10% of locally aggressive RCC cases presented with inferior vena cava (IVC) tumor thrombus [1]. The incidence of advanced RCC in China is significantly higher than those in western countries [2, 3]. The median survival of untreated RCC patients with IVC tumor thrombus was approximately 5 months, and the 1-year tumor-specific survival rate was about 29% [4]. Surgical resection remains the most effective treatment, which can improve the overall survival rate (OS), in particular, the post-operative 5-year survival rate, which can reach 60% in patients without metastasis [1, 5]. With the evolution of surgical techniques and concepts, changes have taken place in surgical strategies for cancer thrombus. Our hospital took the lead in robot-assisted tumor thrombectomy in China and has proved the safety and effectiveness of this technique [6]. In addition to the height of tumor thrombus, the location of the primary tumor, presence of bland thrombus, establishment of collateral circulation, and IVC wall invasion can also affect the surgical outcome [7–9]. IVC wall invasion by tumor thrombus is an independent risk factor for the poor prognosis of RCC [10]. It has been controversial whether the resection of the involved IVC wall during tumor thrombectomy can improve the prognosis. Research has shown that the 5-year survival rate was only 26% in patients without resection of the involved IVC wall, but reached 57% after the resection [11]. The co-existence of bland thrombus and tumor thrombus in RCC is not uncommon, with a reported incidence of up to 13.9% [12]. Previous studies have shown that caval tumor thrombus accompanied by bland thrombus is more likely to be associated with higher grade of thrombus, more diffuse pre-operative metastasis, lower post-operative survival, and worse peri-operative outcomes [13]. During the operation, patients are at risk of death due to the dislodgement of proximal thrombus. Although robot-assisted IVC thrombectomy has been applied increasingly in clinical settings, it is still impossible to judge the IVC wall invasion and the co-existence of bland thrombus using the robotic arm during surgery.

Therefore, how to judge the wall invasion and the presence of bland thrombus by imaging examinations for surgical strategy development has become a hot research topic. Although some CT and MRI studies have been carried out in this field [14–16], these imaging scans are often performed before surgery when the patients are at high blood coagulation state, and in some cases, the tumor thrombus may progress or new bland thrombus may form after imaging examination or before surgery. In contrast, intraoperative ultrasound can be used to detect the wall invasion and bland thrombus in a real-time and dynamic manner under direct vision. Intraoperative ultrasound provides real-time accurate delineation and infiltration of tumor thrombus and thus has the potential to alter surgical decision-making and management. This benefit has been previously described in small case series and case reports, but comprehensive reviews have been lacking [7, 9, 17, 18]. In recent years, contrast-enhanced ultrasound (CEUS) has become a hot research topic in ultrasound medicine. This technique can display the blood perfusion in tumors in real time and dynamically and thus is known as the third revolution after the availability of real-time two-dimensional ultrasound and color Doppler ultrasound [19]. The microbubbles used as the contrast agent of CEUS have a diameter of only 2.5 μm, far smaller than the diameters of the feeding arteries of tumor thrombus. Thus, they can easily enter the tumor thrombus with the blood flow and reflect the real blood supply inside the thrombus [20]. Recently, laparoscopic intra-operative CEUS has become the focus of RCC diagnosis, especially in the partial nephrectomy. In practice, conventional ultrasound may be more useful over the renal hilum where the vessels are much larger and minor movement artifacts are relatively less important. In contrast, CEUS is not affected by movement artifacts caused by the US probe, and thus may be a better technique than the conventional ultrasound [21–24]. During the intra-operative CEUS, ultrasound is applied directly in the surgical field. The probe is placed directly on the surface of the IVC to detect wall invasion and bland thrombus.

The purpose of this study was to investigate the diagnostic performance of intra-operative CEUS in detecting wall invasion and differentiating bland from tumor thrombus of IVC in patients with RCC undergoing nephrectomy in comparison with intra-operative and post-operative pathological findings.

## Materials and methods

### Patients

This retrospective study was approved by the Institutional Review Board of the Chinese PLA General Hospital (approval no. S2017–100-01). Informed consent was signed by all patients. We retrospectively analyzed the data of 60 patients with RCC with IVC tumor thrombus who were treated in our hospital between October 2017 and March 2019. These patients included 46 males and 14 females, aged 30–81 years, with a median age of 54.6 years. Renal tumors were located on the left side in 22 cases and on the right side in 38 cases (Table 1). Of these 60 patients, 50 patients underwent preoperative enhanced MRI examinations with a clinical routine protocol at a 1.5 T unit, within 10 days before surgery. Three patients with claustrophobia, 3 patients with heart stents implant and 4 patients who were allergic to contrast agent did not undertake enhanced MRI.

**Table 1:** Descriptive clinicopathologic characteristics of 60 patients with renal cell carcinoma and inferior vena cava tumor thrombus

Characteristics	Results
Patients, n	60
Median age, yr (interquartile range)	59.5 (47.8–65.0)
Male/Female (n)	46/14
Mean body mass index, kg/m^2^ (range)	23.8 (17.6–30.5)
Affected kidney (n)	
Left	22
Right	38
Mean tumor size, cm (range)	7.6 (3.2–15.6)
Clinical stage (n)	
T3aN0M0	5
T3bN0M0	37
T3bN0M1	9
T3cN0M1	5
T3bN0M1	4
IVC thrombus classification (n)	
Level I	22
Level II	27
Level III	6
Level IV	5
Mean IVC thrombus length cm (range)	7.9 (4.7–13.6)
Presence of bland thrombus (n)	10
Superior bland thrombus (n)	4
Caudal bland thrombus (n)	6
Surgical strategy during IVC thrombectomy	
Incision of the IVC for thrombectomy (n)	51
IVC Segmental transection (n)	9

### Equipment

The da Vinci™ robotic system and surgical equipment, and the B-K Pro Focus 2202 ultrasound system equipped with B-K 8826 laparoscopic probe, with a frequency range of 4–12 MHz, a contact scope of 9 mm × 33.2 mm, and a sector angle of 36 degrees were used. The fin-like catcher on the probe array can be inserted into a variety of common cannulas. The probe was routinely disinfected before surgery. The probe was moved along the IVC wall at any angle required, without leaving any blind spot. The operation was performed using a single hand, and the steps were simple.

### Ultrasound contrast agent

The second-generation contrast agent SonoVue (Bracco, Milan, Italy) was used. SonoVue is based on stabilized sulphur hexafluoride microbubbles with a mean size of 2.5 μm (Fig. 2). Up to 90% of the microbubbles were smaller than 8 μm with a pH range of 4.5–7.5. After mixing with 5 ml saline, 5 ml contrast agent solution was used each time.

### Intra-operative CEUS of IVC tumor thrombus

In all 60 patients with RCC complicated with IVC tumor thrombus, after IVC was fully mobilized by the da Vinci robotic system, the laparoscopic ultrasound probe was placed directly on the surface of the IVC via the auxiliary trocar. The angle of the probe was adjusted so that it was perpendicular to the surface of IVC. The maximum diameter of the IVC tumor thrombus was measured. The area with suspicious wall invasion (initially detected by two-dimensional ultrasound) was identified; sector scan was performed with this area as a center and a dynamic scan was also performed around the lesion. After the CEUS mode was turned on, 5 ml SonoVue contrast agent was injected via peripheral vein pellet, followed by rapid injection of 5 ml saline. The results were observed for 3 min and the whole CEUS process was recorded and saved.

### Image description and analysis

#### Image readers

Two ultrasonographists with more than 3 years of experience in CEUS-based diagnosis who were blind to any preoperative imaging and clinical data of the patients were asked to read the CEUS images.

#### Reading methods

Before image reading, both Reader 1 and Reader 2 received training on reading sample CEUS images of tumor thrombus intra-operatively obtained from 10 patients, who were not included in the final study group. They were required to independently read the images of these 60 patients to determine the presence (or absence) of wall invasion and bland thrombus.

#### Indicators and definitions for image reading

Tumor thrombus does not invade the IVC wall: a) strip-like contrast media can be seen between the tumor thrombus and the IVC wall; and b) the IVC wall continuity is maintained. Tumor thrombus invades the IVC wall: a) the tumor thrombus is enhanced synchronously with the IVC wall; and b) the IVC wall continuity is lost.

#### Differentiation between tumor thrombus and bland thrombus

Tumor thrombus, contrast agent perfusion can be seen in the IVC thrombus. Bland thrombus, no contrast agent is perfused into the IVC thrombus.

According to the results of intra-operative CEUS, Reader 1 and Reader 2 independently read and judged wall invasion and bland thrombus according to the image characteristics. A third senior ultrasonographist was consulted when the results were inconsistent.

### Intra-operative assessment and postoperative pathology

During surgery, wall invasion was diagnosed if the tumor thrombus could not be dissected easily and vice versa. All the pathological results were obtained within 7 days after the examinations. Pathological sections were read by the same pathologist.

### Statistical analysis

Statistical analysis was performed using SPSS 13.0 software. The maximum diameters of tumor thrombus measured by intra-operative ultrasound are presented as mean ± standard deviations. Measurement data between groups were compared using *t* test, and a *P* value of less than 0.05 was considered statistically significant. The results of diagnostic tests are expressed by sensitivity, specificity, accuracy, positive predictive value, and negative predictive value. Cohen’s kappa coefficient was used to measure the inter-observer agreement for categorical variables.

## Results

**Fig. 1:**
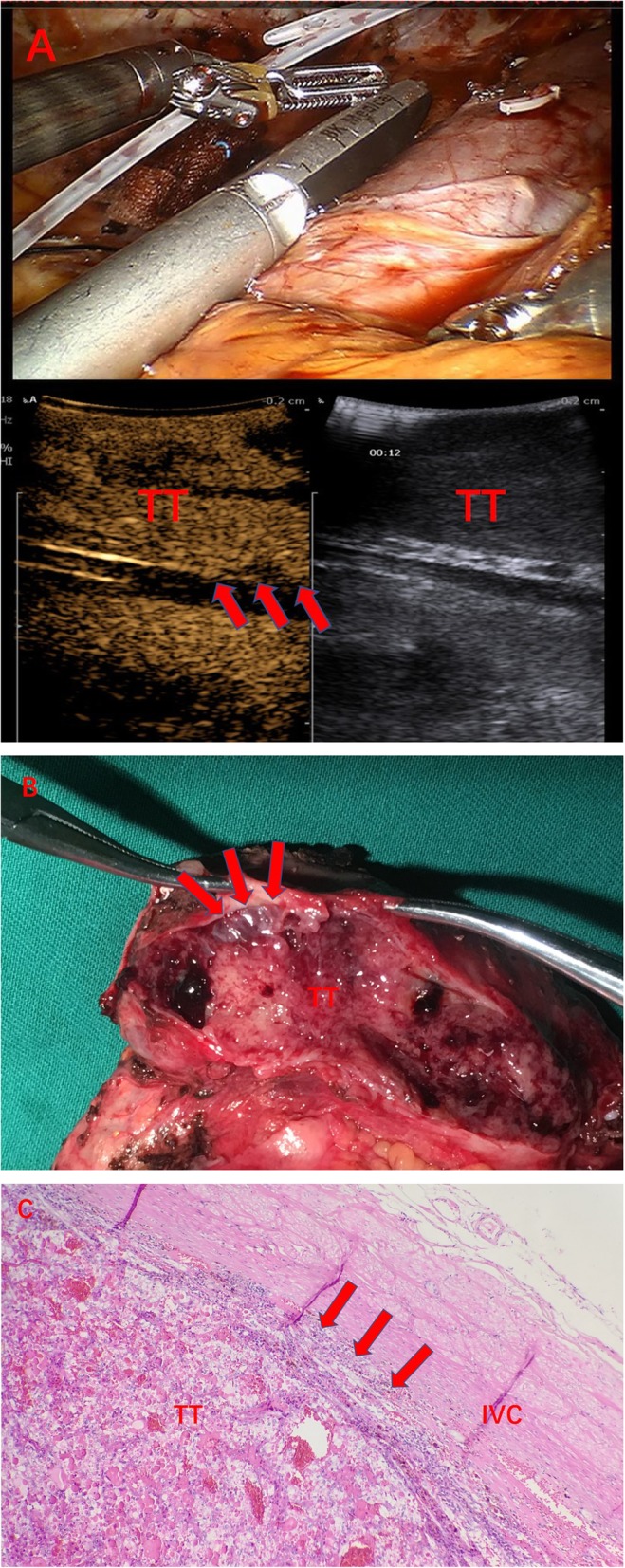
A 47-year-old male patient with a neoplasm of right kidney accompanied by IVC tumor thrombus. He underwent robot-assisted laparoscopic IVC transection. **a** CEUS reveals the tumor thrombus is enhanced synchronously with the IVC wall and the continuity of the IVC wall is poor (arrows), suggesting the presence of wall invasion; **b** postoperative morphology shows the involvement of vascular wall by tumor thrombus (arrows); **c** postoperative pathology shows that the tumor thrombus has invaded the IVC wall (arrows)

**Fig. 2:**
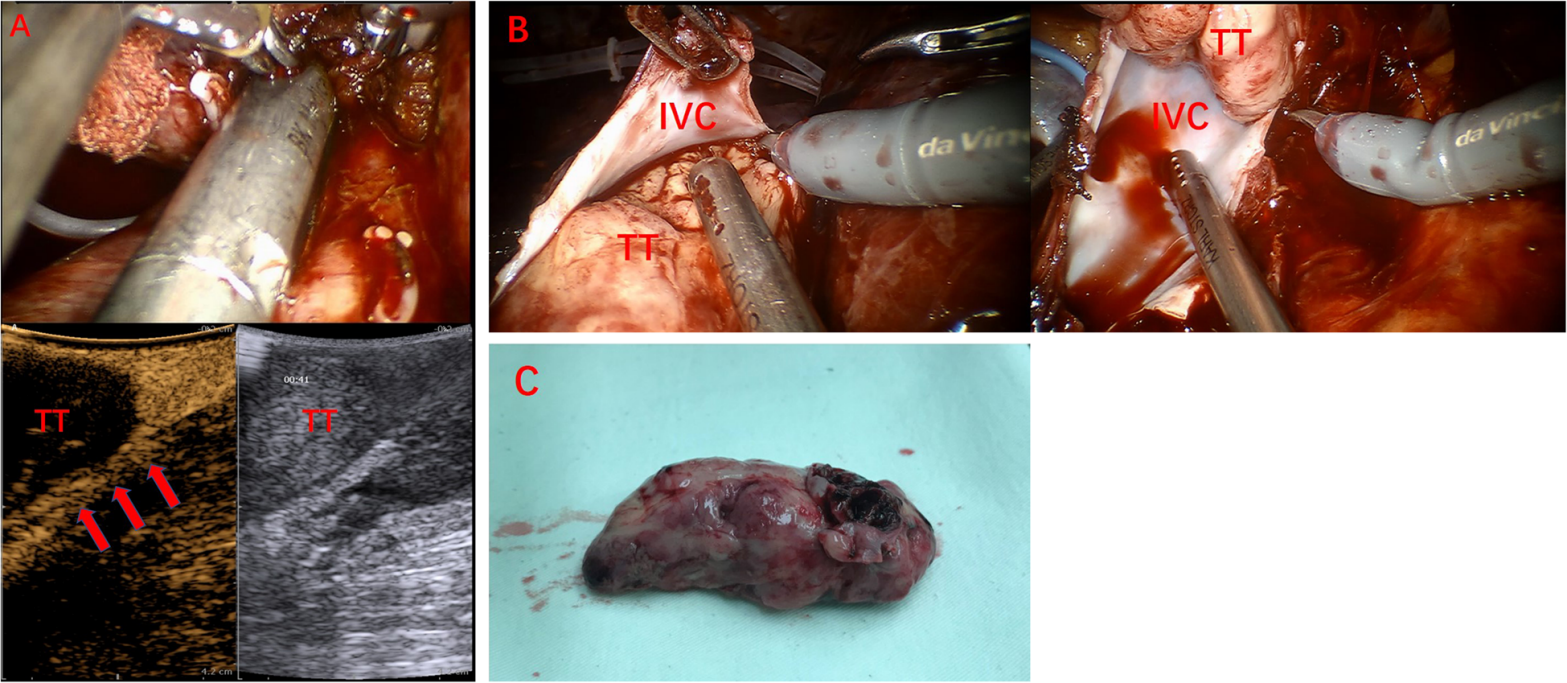
A 50-year-old female patient with a neoplasm of left kidney accompanied by IVC tumor thrombus. She underwent robot-assisted laparoscopic IVC thrombectomy. **a** CEUS reveals the passing of contrast agent between tumor thrombus and IVC wall and the continuity of the IVC wall is good (arrows), suggesting that the IVC wall is not invaded; **b** intra-operative observation indicates that there is no wall invasion; **c** the IVC tumor thrombus is removed en bloc

**Fig. 3:**
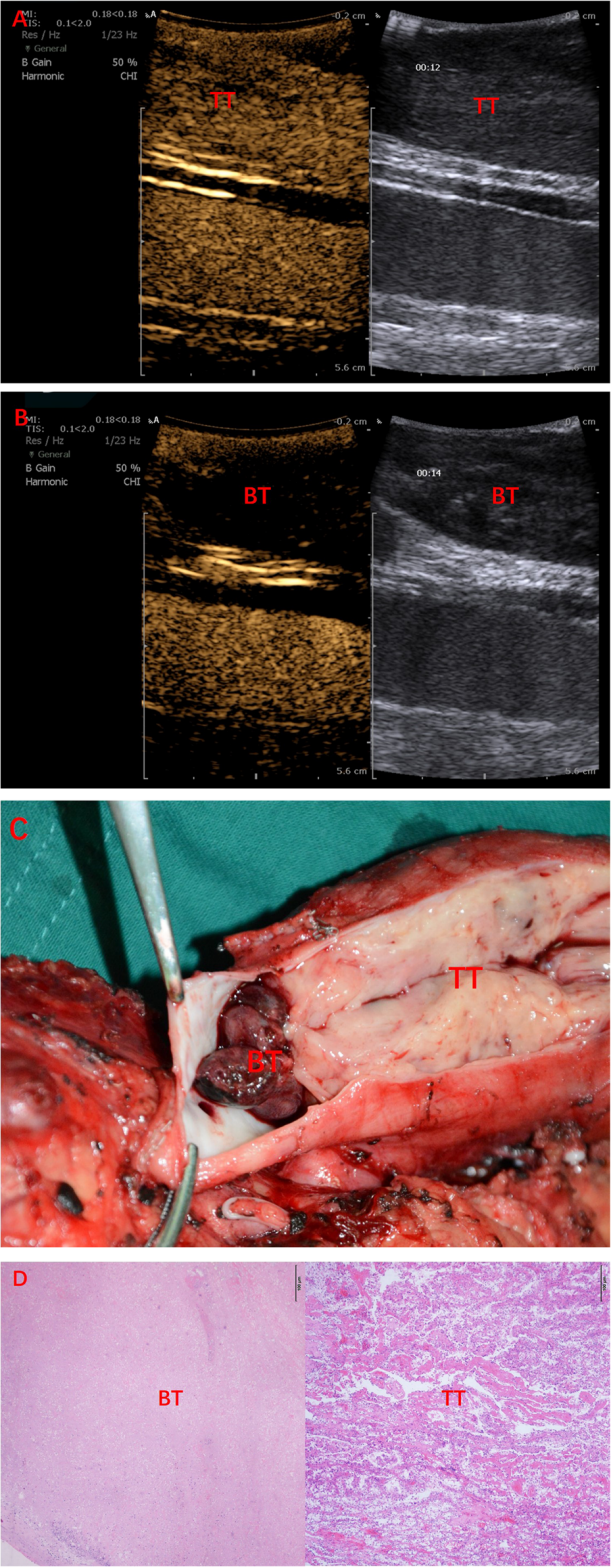
A 53-year-old male patient with a neoplasm of right kidney accompanied by IVC tumor thrombus. He underwent robotic-assisted IVC transection. **a** The tumor thrombus is contrast-enhanced; **b** the bland thrombus is not contrast-enhanced; **c** post-operative morphology shows the co-existence of tumor thrombus and bland thrombus; **d** post-operative pathology reveals the co-existence of tumor thrombus and bland thrombus

All 60 patients with IVC tumor thrombus underwent robot-assisted laparoscopic radical nephrectomy plus IVC thrombectomy, including open IVC thrombectomy in 51 patients, and IVC resection in 9 patients. All the procedures were successfully performed. CEUS images were acquired successfully without any adverse events. According to the clinical and pathological results, wall invasion was found in 29 patients, in whom CEUS showed that the tumor thrombus was enhanced synchronously with the IVC wall, and the continuity of the IVC wall was lost (Fig. 1). No wall invasion was seen in the remaining 31 cases as CEUS showed that the contrast agent could pass between the tumor thrombus and the IVC wall and the continuity of IVC wall was good (Fig. 2, Table 2). The tumor thrombus was accompanied by bland thrombus in 10 cases. Among them, the bland thrombus was located in the head of tumor thrombus in 4 cases and in the tail of tumor thrombus in 6 cases. Contrast-enhanced perfusion was seen in tumor thrombus, but not in bland thrombus (Fig. 3, Table 3).

**Table 2:** Intra-operative CEUS in the diagnosis of wall invasion with surgery and pathology as the reference standard

Intra-operative CEUS diagnosis	Surgical and pathologic diagnosis	
	With wall invasion (*n* = 29)	Without wall invasion(*n* = 31)
Positive	27	2
Negative	2	29

**Table 3:** Intra-operative CEUS in the diagnosis of bland thrombus with surgery and pathology as the reference standard

Intra-operative CEUS diagnosis	Surgical and pathologic diagnosis	
	With bland thrombus (*n* = 10)	Without bland thrombus (*n* = 50)
Positive	10	2
Negative	0	48

The sensitivity, specificity, accuracy, positive predictive value, and negative predictive value of intra-operative CEUS were 93.1, 93.5, 93.3, 93.1 and 93.5%, respectively, in the diagnosis of wall invasion (Table 4) and 100, 96.0, 96.7, 83.3, and 100%, respectively, in the diagnosis of bland thrombus (Table 5). There were excellent inter-observer agreements for determining IVC wall invasion and combining bland thrombus with kappa coefficients of 0.90 and 0.97.

**Table 4:** Diagnostic performance of intra-operative CEUS in the diagnosis of wall invasion with surgery and pathology as the reference standard

	Reader 1	Reader 2	Final results
Sensitivity	96.6% (0.82–1.0)	89.7% (0.73–0.98)	93.1% (0.77–0.99)
Specificity	90.3% (0.74–0.98)	96.8% (0.83–1.0)	93.5% (0.79–0.99)
Accuracy	93.3% (0.84–0.98)	93.3% (0.84–0.98)	93.3% (0.83–0.98)
Positive predictive value	90.3% (0.76–0.96)	96.3% (0.79–0.99)	93.1% (0.78–0.98)
Negative predictive value	96.6% (0.80–0.99)	90.9% (0.77–0.97)	93.5% (0.79–0.98)

95% confidence intervals are indicated in brackets

**Table 5:** Diagnostic performance of intra-operative CEUS in the diagnosis of bland thrombus with surgery and pathology as the reference standard

	Reader 1	Reader 2	Final results
Sensitivity	100% (0.69–1.0)	100% (0.69–1.0)	100% (0.69–1.0)
Specificity	98.0% (0.89–0.99)	94.0% (0.83–0.99)	96.0% (0.86–0.99)
Accuracy	98.3% (0.92–1.0)	95.0% (0.89–1.0)	96.7% (0.91–0.99)
Positive predictive value	90.9% (0.59–0.99)	76.9% (0.52–0.91)	83.3% (0.56–0.95)
Negative predictive value	100% (0.83–1.0)	100% (0.83–1.0)	100% (0.83–1.0)

95% confidence intervals are indicated in brackets

Of the 50 patients undergoing preoperative enhanced MRI examinations within 10 days before surgery, contact of the IVC thrombus to or rupture of the vessel wall were defined as wall invasion. The tumor thrombus was enhanced and the bland thrombus was not enhanced. The sensitivity, specificity, accuracy, positive predictive value, and negative predictive value of preoperative enhanced MRI were 92.3, 91.7, 92, 92.3 and 91.7%, respectively, in the diagnosis of wall invasion and 75, 95.2, 92, 75 and 95.2%, respectively, in the diagnosis of bland thrombus (Table 6). In 2 cases, slight progression was observed intumor thrombus, and new tiny bland thrombus formed in 2 cases after enhanced MRI examination and before surgery.

**Table 6:** Diagnostic performance of intra-operative CEUS (*n* = 60) and enhanced MRI (*n* = 50) in the diagnosis of wall invasion and bland thrombus of RCC with IVC tumor thrombus

	IVC wall invasion		Bland thrombus	
Imaging diagnosis	Enhanced MRI	Intra-operative CEUS	Enhanced MRI	intra-operative CEUS
Sensitivity	92.3% (0.75–0.99)	93.1% (0.77–0.99)	75% (0.35–0.97)	100% (0.69–1.0)
Specificity	91.7% (0.73–0.99)	93.5% (0.79–0.99)	95.2% (0.84–0.99)	96.0% (0.86–0.99)
Accuracy	92% (0.81–0.98)	93.3% (0.83–0.98)	92% (0.81–0.98)	96.7% (0.91–0.99)
Positive predictive value	92.3% (0.75–0.99)	93.1% (0.78–0.98)	75% (0.35–0.97)	83.3% (0.56–0.95)
Negative predictive value	91.7% (0.73–0.99)	93.5% (0.79–0.98)	95.2% (0.84–0.99)	100% (0.93–1.0)

95% confidence intervals are indicated in brackets

The maximum anterior-posterior diameter of the tumor thrombus was 40.12 mm ± 7.32 mm in the wall invasion group and 32.10 mm ± 6.12 mm in the non-invasion group, with significant difference (*P*<0.05), suggesting that the risk of wall invasion increased with the increase of the anterior-posterior diameter of tumor thrombus. In two patients with wall invasion confirmed by intra- and post-operative pathology, CEUS did not detect the synchronous enhancement of the tumor thrombus and the IVC wall due to lack of blood flow signals in tumor thrombus, resulting in misdiagnoses. In another two cases, a diagnosis of wall invasion was made, but post-operative pathology showed that the tumor thrombus did not invade the IVC wall. In fact, the anterior-posterior diameter of tumor thrombus in these two cases was 42.78 mm and 44.23 mm, respectively. The tumor thrombus grew in an expansive way, and they squeezed but did not invade the IVC wall. The IVC wall became thinner due to the compression, which led to the misdiagnosis by intra-operative CEUS.

**Fig. 4:**
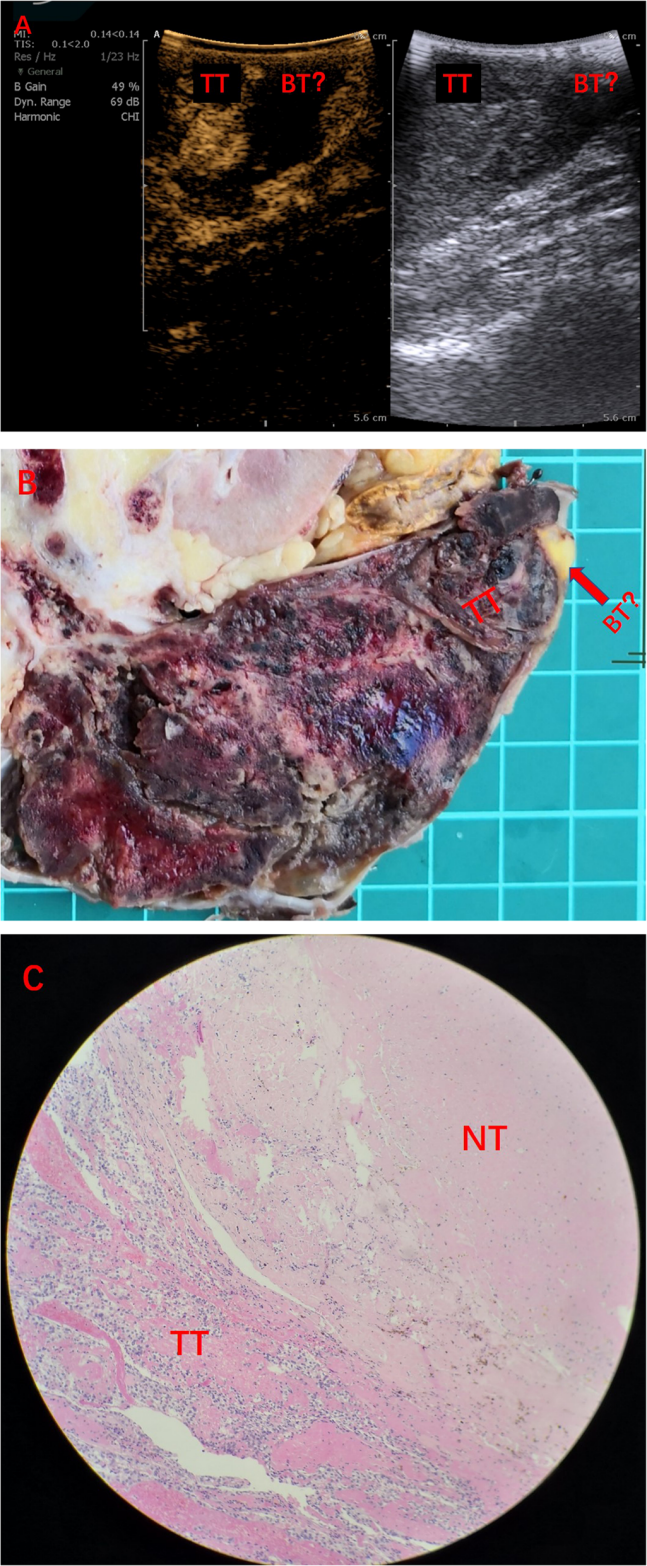
A 55-year-old female patient with a neoplasm of right kidney accompanied by IVC tumor thrombus. She underwent robot-assisted IVC transection. **a** Intra-operative contrast-enhanced ultrasound (CEUS) reveals that there is no perfusion area on the head side of the tumor thrombus, and co-existence of tumor thrombus and bland thrombus? **b** Post-operative morphology shows yellow tissue on the head side of the tumor thrombus (arrows). **c** Post-operative pathology reveals necrotic tissue on the head side of tumor thrombus. IVC = inferior vena cava, TT = tumor thrombus, BT = bland thrombus, NT = necrosis

The co-existence of tumor thrombus and bland thrombus, as confirmed by intra- and post-operative pathology, in 10 patients was correctly diagnosed by intra-operative CEUS. However, the necrotic tissues in the head of tumor thrombus in 2 cases were misdiagnosed as bland thrombus by intra-operative CEUS (Fig. 4).

## Discussion

RCC complicated with ICC tumor thrombus is a T_3_ advanced renal cancer, which used to be a contraindication for laparoscopic surgery. In 2011, robot-assisted laparoscopic radical nephrectomy plus IVC thrombectomy was first described by Abaza [24]. With the advances in the da Vinci laparoscopic robotic surgery, the robot-assisted laparoscopic IVC thrombectomy has become mature [7–9]; however, the handling and reconstruction of IVC are challenging during this procedure [7]. The IVC wall invasion and the presence of bland thrombus in the long distal segment of IVC are key indications for IVC transection [25]. In addition, a lethal factor in IVC thrombectomy is pulmonary embolism caused by the proximal bland thrombus shedding [26]. Compared with patients without accompanying bland thrombus, patients with the co-existing bland thrombus present with more obvious clinical symptoms, greater intra-operative blood loss, higher rate of blood transfusion, and longer tumor thrombus. Analysis of prognosis showed that patients with the co-existing bland thrombus had significantly inferior progression-free survival and overall survival than those without bland thrombosis during the follow-up period [12, 13]. Therefore, IVC wall invasion and co-existence of bland thrombus are major concerns in robot-assisted laparoscopic radical nephrectomy plus IVC thrombectomy. They can affect surgical decision-making and may also be related to the post-operative survival. Previously, preoperative imaging assessment was mainly performed by CT and MRI.

According to a recent study [14], the sensitivity and specificity of preoperative MRI in detecting IVC wall invasion were 92.3 and 86.4%, respectively. Stern et al. [16] found in multidetector computed tomography (MDCT) images that neovascularization of tumor thrombus resulted in uneven enhancement of tumor thrombus in the arterial phase, suggesting the involvement of IVC wall. Sheth et al. [27] also performed MDCT and found that the arteries feeding the tumor thrombus from adjacent organs in the early arterial phase might predict wall invasion, and cross-sectional images were more useful for observation. Aslam et al. [15] compared seven possible signs and found that the tumor thrombus was enhanced after the use of contrast agent; in addition, the thrombus penetrating the blood vessel wall was clearly visible, resulting in tumor signals detected on both sides of the vascular wall. This is the most reliable sign of vascular wall involvement on MRI. The enhancement features of IVC tumor thrombus accompanied by bland thrombus on MRI were as follows: the tumor thrombus was enhanced and the bland thrombus was not enhanced; the signal intensities were mixed in most cancer thrombus and homogeneous in bland thrombus [12]. Thus, although MRI and MDCT have certain diagnostic values in detecting IVC wall invasion and the accompanying bland thrombus, their applications in clinical settings should be cautious. For instance, patients with claustrophobia and those who have been placed with heart stents or cochlear implants should not undertake MRI. The radiation of MDCT and the allergic reactions to contrast agent should also be taken into consideration. Notably, these imaging methods are performed before surgery; in some cases, the tumor thrombus may progress or new bland thrombus may form after imaging examination and before surgery.

Laparoscopic surgeons cannot directly palpate organs, thus lacking direct tactile feedback. The height of tumor thrombus, invasion of IVC, and presence of bland thrombosis cannot be judged by the touch of robotic arm. As a combination of intra-operative ultrasound and laparoscopy, laparoscopic ultrasound [28] can scan the lesions directly, thus shortening the contact distance between ultrasound probe and organs and enabling real-time dynamic monitoring. Laparoscopic ultrasound has been widely used in surgery [29, 30], especially in urologic surgery, mainly for removal of endogenous and renal hilar tumors [31, 32]. CEUS is a new sonographic technique that injects contrast agents via peripheral veins into the body. It can significantly increase the visibility of low-speed blood flow and clearly visualize the signals of small blood flow containing contrast agents and the tissue microvascular perfusion [33]. Ultrasound contrast agent SonoVue is a real blood pool tracer. Unlike CT and MRI enhancers, SonoVue always circulates in the blood vessel, distributes in the whole blood through the pulmonary circulation, and is discharged through breathing. It is safe, harmless, non-toxic, and convenient. With an injection dosage much less than that of CT contrast agent, it offers high time-resolution and can monitor microcirculation perfusion dynamically and in a real-time manner [34]. Laparoscopic intra-operative CEUS has been used in robot-assisted laparoscopic partial nephrectomy. Its real-time dynamic intra-operative scanning can accurately detect the location, size, scope, depth, and blood supply of the tumors, providing effective parameters for the partial nephrectomy. Thus, it ensures the safety of surgery, reduces the residual and recurrent tumors, and lowers the incidence of postoperative complications [20, 21].

In the present study, we applied the intra-operative CEUS for the first time in robot-assisted laparoscopic IVC thrombectomy. It allows the observation of the enhancement of IVC tumor thrombus in a real-time manner and can visualize the blood perfusion inside the thrombus and on vascular wall sensitively and intuitively; thus, it can objectively reflect the presence (or absence) of the blood supply of tumor thrombus, the route of blood supply, the synchronous enhancement of the tumor thrombus, and even the inferable composition of tumor thrombus. On one hand, when the tumor thrombus invades the IVC wall, the venous wall will be enhanced synchronously with the tumor thrombus on CEUS. If the tumor thrombus does not invade the IVC wall, the passing of contrast agent between the cancer thrombus and the venous wall can be clearly visualized due to the high sensitivity of CEUS in detecting blood flow. On the other hand, the composition and blood supply characteristics of the tumor thrombus are completely different from those of the bland thrombus in IVC, which provides a basis for differentiating these two thrombus by CEUS. The components of IVC bland thrombus include platelets, white blood cells, red blood cells, and cellulose. As there is neither active substance nor blood supply, the IVC tumor thrombus is not enhanced on CEUS.

As shown in the present study, intra-operative CEUS could sensitively reflect the blood perfusion of IVC thrombus, the wall invasion by tumor thrombus, and the presence of bland thrombus. Its high resolution allows the operator to learn the blood supply of tumor thrombus in a real-time and intuitively manner, which reminds the doctor to take corresponding measures timely to ensure a successful operation.

Our study had some limitations. First, as a retrospective study, it might have potential selection bias; second, this is a single-center study with a relatively small sample size.

## Conclusion

This study indicates that CEUS in robot-assisted IVC thrombectomy can detect wall invasion and differentiate bland thrombus from tumor thrombus, thus offering real-time information to the operator during surgery for RCC.

## Data Availability

The datasets used and/or analysed during the current study are available from the corresponding author at reasonable request.

## Abbreviations

BT: bland thrombus
CEUS: Contrast-enhanced ultrasound
CT: Computed tomography
IVC: Inferior vena cava
MRI: Magnetic resonance imaging
NT: necrosis
RCC: Renal cell carcinoma
TT: tumor thrombus
